# Bone morphogenetic protein-15 in follicle fluid combined with age may differentiate between successful and unsuccessful poor ovarian responders

**DOI:** 10.1186/1477-7827-10-116

**Published:** 2012-12-26

**Authors:** Yan-Ting Wu, Ting-Ting Wang, Xi-Jing Chen, Xiao-Ming Zhu, Min-Yue Dong, Jian-Zhong Sheng, Chen-Ming Xu, He-Feng Huang

**Affiliations:** 1 Department of Reproductive Endocrinology, Women’s Hospital, School of Medicine, Zhejiang University, Hangzhou, China; 2 Department of Pathology and Pathophysiology, School of Medicine, Zhejiang University, Hangzhou, China; 3 Key Laboratory of Reproductive Genetics, Zhejiang University, Ministry of Education, Hangzhou, China

**Keywords:** Poor response, IVF-ET, BMP-15, Oocyte, Age, Retrospective study

## Abstract

**Background:**

The counselling of poor ovarian responders about the probability of pregnancy remains a puzzle for gynaecologists. The aim of this study was to optimise the management of poor responders by investigating the role of the oocyte-derived factor bone morphogenetic protein-15 (BMP-15) combined with chronological age in the prediction of the outcome of in-vitro fertilisation-embryo transfer (IVF-ET) in poor responders.

**Methods:**

A retrospective study conducted in a university hospital. A total of 207 poor ovarian responders who reached the ovum pick-up stage undergoing IVF/intracytoplasmic sperm injection (ICSI) with three or fewer follicles no less than 14 mm on the day of oocyte retrieval were recruited from July 1, 2008 to December 31, 2009. Another 215 coinstantaneous cycles with normal responses were selected as controls. The BMP-15 levels in the follicular fluid (FF) of the 207 poor responders were analysed by western blot. Based on the FF BMP-15 level and age, poor responders were sub-divided into four groups. The main outcome measures were the FF BMP-15 level, implantation rate, pregnancy rate, and live birth rate.

**Results:**

The implantation rate (24.2% vs. 15.3%), chemical pregnancy rate (40% vs. 23.7%), clinical pregnancy rate (36.5% vs. 20.4%) and live birth rate (29.4% vs. 15.1%) in the high BMP-15 group were significantly higher than those in the low BMP-15 group. Furthermore, poor responders aged less than or equal to 35 years with a higher FF BMP-15 level had the best implantation, pregnancy and live birth rates, which were comparable with those of normal responders.

**Conclusions:**

Our study suggests a potential role of BMP-15 in the prediction of the IVF outcome. A high FF BMP-15 combined with an age less than or equal to 35 years may be used as a potential indicator for repeating IVF cycles in poor ovarian responders.

## Background

Poor ovarian response is a major challenge in in-vitro fertilisation-embryo transfer (IVF-ET). Because many women today are postponing pregnancy and the average age of IVF patients has increased correspondingly, poor response is not a rare occurrence in ovarian stimulation and has become part of the daily clinical management for clinicians in IVF centres [1]. According to our clinical experience, few poor responders would like to give up an initiated cycle with most wanting to know the chance of conception during the next cycle if they were not pregnant. Appropriate counseling would help patients decide whether to continue or stop IVF treatment to avoid both unnecessary economic losses and health risks. Many hormonal and ultrasound markers, such as age, basal serum follicle stimulating hormone (FSH) level, serum anti-Mullerian hormone levels and the number of antral follicles, have been developed to predict the cycle success or identify patients at high risk of poor response before ovulation induction [2-4]. However, there is still no ideal and consistent predictive marker of IVF outcome for poor ovarian responders.

The oocyte has a central role in regulating follicle growth and ovulation by producing oocyte-derived factors [5]. These oocyte-derived factors, such as growth differentiation factor-9 (GDF-9) and bone morphogenetic protein-15 (BMP-15), may determine the sensitivity of follicles to FSH and the selection of follicles to the dominant follicles that continue growth to the pre-ovulatory stage [6]. BMP-15, one of the most important oocyte-derived factors, plays an essential role in female fertility [5]. Variants of the BMP-15 gene have been associated with premature ovarian failure and ovarian hyperstimulation syndrome in women [7,8]. BMP-15 is a key mitogenic factor for somatic cells and a stimulator of granulosa cell proliferation [9]. Importantly, our previous study demonstrated a correlation of the BMP-15 level in follicular fluid (FF) with oocyte quality and subsequent embryo development [10].

Female age has been well established to be the most significant factor influencing clinical IVF outcome, associated with reduced oocytes and increased early pregnancy loss. Herein, we hypothesized that the BMP-15 levels in FF combined with maternal age might be a candidate marker for the prediction of IVF outcomes in poor responders. In the present study, we investigated the relationship among FF BMP-15 level, maternal age and outcome in poor ovarian responders.

## Methods

### Patients

The protocol was approved by the Institutional Review Board of Zhejiang University School of Medicine. Informed consents were obtained from all participants. Poor responders were defined as women having 3 or fewer follicles with a diameter ≥ 14 mm on the day of oocyte retrieval. Normal responders were defined as those having 4 to 12 such follicles [11]. The exclusion criteria were endometriosis, hyperprolactinemia and polycystic ovarian syndrome. Women who are in their 40s have a significantly reduced chance of achieving pregnancy and delivery by means of IVF compared with women who are in their 30s. Thus we only included women who were less than 40 years of age in this study. A total of 207 infertile women undergoing IVF/ICSI treatment were recruited from July 1, 2008 to December 31, 2009 at the Centre for Reproductive Medicine, Women’s Hospital, Zhejiang University School of Medicine, China. Moreover, 215 age-matched normal responders were recruited as controls.

### Protocol and collection of follicular fluid

Pituitary desensitisation was commenced during the luteal phase preceding IVF treatment with the gonadotropin releasing hormone agonist, triptorelin, at 0.1 mg per day (Decapeptyl; Ferring, Malmo, Sweden). Follicle growth was stimulated from day 3 of the menstrual cycle by injecting recombinant FSH (Gonal-F; Serono, Abonne, Switzerland) at 300IU/d for the first 2 days, followed by the administration of rFSH at 150–225 IU/d (for women with basal FSH<10 or aged ≤35 years) or 225–300 IU/d (for women with basal FSH>10 or aged >35 years) with adjustment as necessary according to the serum estradiol levels and transvaginal ultrasonography. A total of 10000 IU of urinary human chorionic gonadotrophin (uHCG, Livzon, China) was administered when the leading follicle reached 18–22 mm in diameter, as detected by ultrasound. Oocyte retrieval was performed 36 hours later under transvaginal ultrasound guidance.

Clean follicular fluid was collected by trans-vaginal ultrasound-guided puncture and aspiration of 18–20 mm follicles. Only the fluid from the first aspirated follicle of each patient was carefully collected to avoid blood contamination. FF samples were centrifuged at 2647*g* for 10 min and stored at −70°C until the assay.

According to the cause of infertility, fertilisation was conducted by IVF or ICSI, as determined by our clinicians independently. Fertilisation was considered normal when the oocytes contained 2 pronuclei. The embryo transfer was performed on day 2 or 3, depending on the embryo quality and quantity. A pregnancy test was performed at 2 weeks and an ultrasound examination at 4 weeks after the embryo transfer. Each pregnant woman was followed up until pregnancy termination. The implantation rate was defined as the percentage of embryos transferred that implanted and developed to the stage of ultrasound documented fetal heartbeat. Chemical pregnancy was defined as a positive result for pregnancy by a blood HCG test, and clinical pregnancy was defined as the presence of an intrauterine gestational sac on the trans-vaginal ultrasound examination.

### Analysis of BMP-15 level in FF

BMP-15 protein level in FF was analysed by Western blotting as previously described [9,10]. Briefly, 400 nanoliters of FF was diluted with 0.4 μl of lysis buffer (20 mM Tris pH 7.4, 150 mM NaCl, 2 mM ethylenediaminetetraacetic acid, 0.1% sodium dodecyl sulphate [SDS], 1% Triton X-100, 0.5% sodium deoxycholate, 10% glycerol) and electrophoresed on 12% SDS polyacrylamide gel electrophoresis under reducing conditions. The separated proteins were transferred onto nitrocellulose membranes, which were then blocked for 1 h in Tris-buffered saline (TBS) containing 5% non-fat dry milk. Subsequently, the membranes were incubated with an antibody against BMP-15 (1:5000, Santa Cruz Biotechnology, Santa Cruz, CA, USA) in TBS-Tween 20 and then incubated with anti-rabbit IgG (1:40000, Santa Cruz Biotechnology) in TBS-Tween 20. The membranes were washed several times with TBS Tween 20 after each step and visualised with an enhanced chemiluminescence system (Amersham Pharmacia Biotech, Freiburg, Germany). The images were scanned with the Bio-Rad GS800 densitometer (Bio-Rad Laboratories, Hercules, CA, USA) and analyzed using Quantity One software (Bio-Rad). The FFs of three random poor responders were pooled to generate a standard sample, which was included in each blot as a reference to permit comparison between blots. The intensities of individual bands were expressed as arbitrary absorbency units and normalised to the standard sample. The measurement was duplicated for each sample, and the values were averaged before the statistical analysis.

### Statistical analysis

The statistical analysis was performed with the Statistical Package for the Social Sciences. Continuous variables were presented as medians (ranges) and analysed using the Kruskal-Wallis test or Mann–Whitney-*U* test. The chi-square test and likelihood ratios were used to compare IVF outcomes. For all analyses, the significance was set at *P* < 0.05.

## Results

**Figure 1:**
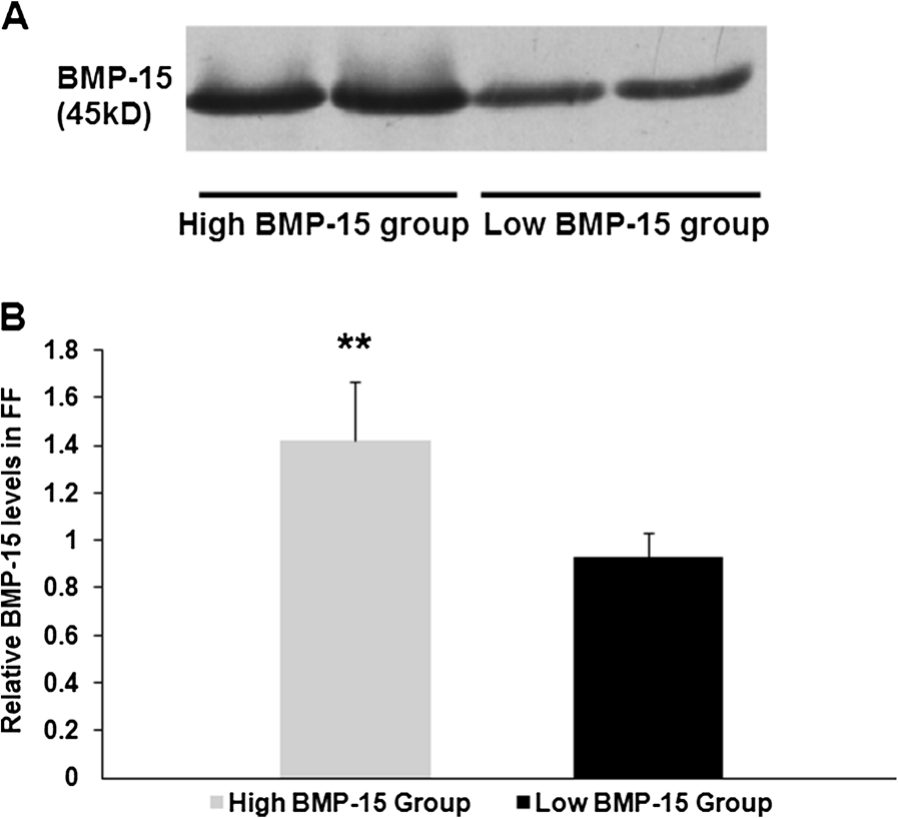
**Relative BMP-15 levels in poor responders.A**: Western blotting analysis of the BMP-15 protein in follicular fluid. The BMP-15 in 0.4 μl of FF following SDS gel electrophoresis, transfer and blotting on a nitrocellulose membrane with a polyclonal antibody. **B**: Comparison of the relative levels of BMP-15 in follicular fluid by western blotting analysis in poor responders. The level of follicular fluid BMP-15 in the high BMP-15 group was significantly higher than that in the low BMP-15 group (**P < 0.001).

A total of 207 poor responders and 207 normal responders were recruited in this study. These women were aged 24–39 years, and their mean age was 34 years. Only the first-puncture, clear FF samples from each patient were analysed to avoid blood contamination. Overall, 207 FF samples from the poor responders were collected. Western blotting analysis showed a single band at approximately 45 kDa, which represented BMP-15, in all 207 poor responder FF samples (Figure 1). The average level of the BMP-15 protein was 1.17 (normalised to the standard sample). Based on the FF BMP-15 levels of the 207 poor responders, they were divided into a high BMP-15 group (higher than the mean value) and a low BMP-15 group (lower than the mean value). As shown in Table 1, age, body mass index (BMI), basal FSH, number of retrieved oocytes, ET cycles and number of transferred embryos were not significantly different between the two groups. However, the implantation rate (25.3% vs. 15%), chemical pregnancy rate (41.5% vs. 23.3%), clinical pregnancy rate (37.8% vs. 21.1%) and live birth rate (30.5% vs. 15.6%) were significantly higher in the high BMP-15 group than in the low BMP-15 group (Table 1, *P*<0.05).

**Table 1:** Comparison of the characteristics and outcomes between women with different follicular BMP-15 levels

**Parameter**	**Low BMP-15 (110 cycles)**	**High BMP-15 (97 cycles)**	***P*value**
*BMP-15 level*	0.95 (0.64–1.12)	1.35 (1.18–2.09)	<0.001^a^
*Age (yrs)*	34 (24–39)	34 (25–39)	0.670^a^
*BMI (kg/m*^*2*^*)*	21.5 (15.8–33.8)	22.0 (15.2–28.0)	0.270 ^a^
*Basal FSH (IU/L)*	7.46 (3.28–18.73)	8.0 (3.19–17.29)	0.137^a^
*No. of oocytes retrieved*	3 (0–7)	3 (0–7)	0.396^a^
*ET cycle/started cycle*	81.8%	84.5%	0.602^b^
*No. of embryos transferred*	2 (0–3)	2 (0–3)	0.477^a^
*Implantation rate/embryo*	15%	25.3%	0.023^b^
*Chemical pregnancy rate/ET cycle*	23.3%	41.5%	0.011^b^
*Clinical pregnancy rate/ET cycle*	21.1%	37.8%	0.016^b^
*Live born rate/ET cycle*	15.6%	30.5%	0.019^b^
*Miscarriage rate*	26.3%	19.4%	0.567^b^

^a^ Values presented as medians (ranges) and compared by the Mann-Whitney-*U* test.

^b^ Values are compared with Chi-square test (likelihood ratio).

According to a maternal age cut-off of 35 years, the high BMP-15 and low BMP-15 groups were further divided into four sub-groups, as shown in Table 2. The number of retrieved oocytes, ET cycles and transferred embryos were comparable among the sub-groups. The low BMP-15 sub-group older than 35 years had the lowest implantation (6.5%) and clinical pregnancy (12.5%) rates. The high BMP-15 sub-group younger than 35 years had the highest live birth rate (38.3%) among the four sub-groups, which was significantly higher than that in the low BMP-15 sub-group older than 35 years (Table 2). Notably, poor responders older than 35 years with either high or low BMP-15 levels had a significantly higher miscarriage rate than high BMP-15 patients aged ≤35 years.

**Table 2:** Comparison of the characteristics and IVF outcomes among poor responders subdivided by FF BMP-15 level and age

**Parameter**	**Low BMP-15 (110)**		**High BMP-15 (97)**		**P value**
	**Group 1**	**Group 2**	**Group 3**	**Group 4**	
	**(Age≤35, n=67)**	**(35<Age<40, n=43)**	**(Age≤35, n=56)**	**(35<Age<40, n=41)**	
*No. of oocytes retrieved*	3 (0–7)	3 (0–7)	3 (0–7)	3 (0–6)	0.300^a^
*ET cycle/started cycle*	86.6%	74.4%	83.9%	85.4%	0.415^b^
*No. of embryos transferred*	2 (0–3)	2 (0–3)	2 (0–3)	2 (0–3)	0.816^a^
*Implantation rate/embryo*	20.4%	6.5%	28.9%	20.9%	0.011^c^
					<0.001^d^
					0.015^e^
*Chemical pregnancy rate/ET cycle*	27.6%	15.6%	44.7%	37.1%	0.005^d^
					0.044^e^
*Clinical pregnancy rate/ET cycle*	25.9%	12.5%	40.4%	34.3%	0.005^d^
					0.033^e^
*Live birth rate/ET cycle*	20.7%	6.25%	38.3%	20%	0.054^c^
					0.001^d^
*Miscarriage rate*	20%	50%	5.3%	41.7%	0.035^d^ 0.012^f^

^a^ Values presented as medians (ranges) and compared across four groups by the Kruskal-Wallis tests.

^b^ Comparison across four groups (likelihood ratio).

^c^ Group 1 vs. Group 2 (likelihood ratio).

^d^ Group 2 vs. Group 3 (likelihood ratio).

^e^ Group 2 vs. Group 4 (likelihood ratio).

^f^ Group 3 vs. Group 4 (likelihood ratio).

Table 3 demonstrates the cycle details and outcomes of poor responders aged ≤ 35 with high FF BMP-15 levels, and those of normal responders. The number of retrieved oocytes, ET cycles and number of transferred embryos in the normal responders were significantly higher than those in the poor responders. However, there were no significant differences in the implantation rate (28.9% vs. 24.8%), chemical pregnancy rate (44.7% vs. 47.1%), clinical pregnancy rate (40.4% vs. 45.6%) and live birth rate (38.3% vs. 37.4%) between the two groups.

**Table 3:** **Comparison of the characteristics and IVF outcomes between poor responders aged ≤35 years with high FF BMP-15 levels and normal responders**

**Parameter**	**Poor responders aged≤35 with high BMP-15 (56 cycles)**	**Normal responders (215 cycles)**	***P*value**
*No. of oocytes retrieved*	3 (0–7)	9 (3–21)	<0.001^a^
*ET cycle/started cycle*	83.9%	95.8%	0.004^b^
*No. of embryos transferred*	2 (0–3)	2 (0–3)	<0.001^a^
*Implantation rate/embryo*	28.9%	24.8%	0.436 ^b^
*Chemical pregnancy rate/ET cycle*	44.7%	47.1%	0.756^b^
*Clinical pregnancy rate/ET cycle*	40.4%	45.6%	0.516^b^
*Live birth rate/ET cycle*	38.3%	37.4%	0.907^b^
*Miscarriage rate*	5.3%	18.7%	0.121^b^

^a^ Values presented as medians (ranges) and compared by the Mann-Whitney-*U* test.

^b^ Values are compared with Chi-square test (likelihood ratio).

## Discussion

The quality and quantity of the retrieved oocytes are the major factors affecting IVF outcomes. For poor responders, numerous ovarian reserve tests have been developed to predict the response to ovarian stimulation [1,2,11,12]. Methods using endocrine and ultrasound markers for assessing the quantity of follicles are widely performed. However, few methods have been established to evaluate the quality of the oocytes. In the present study, we found a role for the oocyte-derived factor, BMP-15, in predicting IVF outcome in poor responders, which may facilitate the further management of these patients.

First, we divided the 207 poor responders into a high BMP-15 group and a low BMP-15 group according to their FF BMP-15 levels. Both groups were comparable in terms of age, but the implantation and pregnancy rates were significantly higher in the high BMP-15 group. These data suggest that FF BMP-15 may be an effective marker of oocyte quality and IVF performance independent of age. This result is consistent with our previous study, which showed that oocytes retrieved from follicles with a high FF BMP-15 level had higher fertilisation rates and a better quality of developed embryos [10].

The potential role of BMP-15 in oocyte development competence and pregnancy outcome is well grounded. In ovary, it is exclusively produced by oocytes [5]. BMP-15 has been shown to stimulate undifferentiated granulosa cell proliferation, regulate granulosa cell differentiation [9], and inhibit FSH action by suppressing the sensitivity of granulosa cells to FSH stimulation [13]. Importantly, BMP-15 is also involved in the selection of the dominant follicle and in oocyte maturation by cooperating with FSH [14]. Moreover, BMP-15 may contribute to the maintenance of the low incidence of cumulus cell apoptosis by establishing a localized gradient [15], and it induces cumulus cell expansion by enhancing the expression of epidermal growth factor-like growth factor [16]. Interestingly, BMP-15 can promote glycolysis in cumulus cells for oocyte development as mammalian oocytes are unable to initiate glycolysis [5,17].

Many reports have identified the hormones and other factors in FF and their roles in evaluating oocyte quality. The FF concentrations of hormones, prolactin, interleukin-1, inhibin B, tumour necrosis factor, free fatty acids and dehydroepiandrosterone have been associated with the rates of normal fertilisation and good embryo development and the quality of the embryos [10,18,19]. However, their predictive accuracy and sensitivity is very limited, and some reports did not replicate these findings [20,21]. In contrast to all of the factors mentioned above, BMP-15 is oocyte-derived, indicating that it may be a more direct and valuable marker than others for predicting oocyte quality. Unsurprisingly, therefore, poor responders with a higher FF BMP-15 level had better IVF outcomes, including a higher live birth rate.

Ageing is well-known to reduce the biological capacity of a woman to reproduce. In this study, we found that poor responders with both low FF BMP-15 and age >35 years had the worst IVF outcomes. Meanwhile, young responders with high FF BMP-15 showed a better outcome. Even in the high BMP-15 group, increased maternal age was also associated with a significantly greater miscarriage rate. These clinical consequences may be due to the increase in the incidence of oocyte meiotic errors with age [22]. Notably, the miscarriage rate of women with high FF BMP-15 and aged less than or equal to 35 years was just 5.3%.

Because IVF is an invasive and expensive treatment, informing poor responders when to give up further IVF cycles is important. The combination of BMP-15 and age appears more accurate than any single marker in predicting the clinical outcome, which may help poor responders decide whether to continue with further attempts. Moreover, for poor responders with one or two mature follicles, proceeding to IVF may represent their best chance for a successful pregnancy. Conversion to intrauterine insemination or cancelling the current cycle and making another attempt later does not improve outcome [23]. Our and other studies suggest that proceeding to IVF may still be the best choice for these patients, especially for those younger than 35 years and with a higher BMP-15 in FF. However, patients with both low FF BMP-15 levels and aged >35 years should be adequately warned of the possibility of the worst pregnancy outcome. We did not determine the value of FF BMP-15 in another IVF cycle in these patients, and whether the BMP-15 level would be changed in the following IVF cycle with adjusted stimulation protocols remains unknown. Further studies using large samples sizes should be undertaken before a clear conclusion is made.

## Conclusion

A high FF BMP-15 level combined with a young age confers a better pregnancy opportunity. Therefore, further IVF attempts should be encouraged for those poor responders with high FF BMP-15 and young age. However, for older poor responders with low FF BMP-15, a forewarning of a poor pregnancy outcome may help the couple to change to other, more appropriate treatments and avoid the cost and futility of repeated IVF cycles.

## Abbreviations

BMP-15: Bone morphogenetic protein-15; FF: Follicular fluid; IVF-ET: In-vitro fertilisation-embryo transfer; ICSI: Intracytoplasmic sperm injection; FSH: Follicle stimulating hormone; LH: Luteinising hormone; HCG: Human chorionic gonadotrophin; SDS: Sodium dodecylsulphate; TBS: Tris-buffered saline; BMI: Body mass index.

## Competing interests

The authors declare that they have no competing interests.

## Authors’ contributions

YTW conducted the data analysis and drafted the manuscript. TTW performed the western blotting and statistical analysis. HFH designed the study and validated the data. XMZ supervised the patient diagnosis. XJC and CMX recruited the samples. MYD and JZS revised the manuscript. All authors read and approved the final manuscript.
